# Prioritizing Global Public Health Investments for COVID-19 Response in Real Time: Results from a Delphi Exercise

**DOI:** 10.1089/hs.2021.0142

**Published:** 2022-04-22

**Authors:** Patrick L. Osewe, Michael A. Peters

**Affiliations:** Patrick L. Osewe, MD, MPH, is Chief of Health Sector Group, Asian Development Bank, Manila, Philippines.; Michael A. Peters, MSPH, PhD, was a Consultant, Asian Development Bank, Manila, Philippines. He is now Associate Faculty, Department of International Health, Johns Hopkins Bloomberg School of Public Health, Baltimore, MD.

**Keywords:** COVID-19, Infectious diseases, Agenda setting, Delphi technique

## Abstract

In the first months of the COVID-19 pandemic, there was a lack of guidance on how to channel the unprecedented amount of health financing toward the pandemic response. We employed a multistep, interactive Delphi process to reach consensus on a “menu” of priority COVID-19 response interventions. In all, 27 health security experts—representing national governments, bilateral and multilateral organizations, academia, technical agencies, and nongovernmental organizations—participated in the exercise. The experts rated 11 technical investment areas and 37 interventions on a 5-point scale in terms of their importance to COVID-19 response. Initial findings were discussed at a virtual meeting where experts suggested modifications. A group of 19 experts then rated a revised list of 11 technical areas and 39 interventions. Consensus was defined as at least 80% of experts agreeing on the importance of a technical area or intervention; stability of scores across the rounds was identified using Wilcoxon matched-pairs and unpaired signed rank tests. Between the initial and final menu, 3 technical areas and 7 interventions were slightly modified, 3 interventions were added, and 1 intervention was removed. Consensus was reached on all 11 technical areas and 35 of the final 39 interventions, and between 34 and 37 interventions were stable across rounds depending on the test used. In this exercise, the health security experts agreed that COVID-19 response financing should prioritize interventions that enhance a country's capacity to test, trace, and treat high-risk populations. Simultaneously, supportive systems (eg, risk communication, community engagement, public health infrastructure, information systems, policy and coordination, workforce capacity, other social protections) should be developed to ensure that nonpharmaceutical and medical interventions can maximize the effectiveness of these systems.

## Introduction

For years, public health experts have been warning of the potentially devastating effects of a new virulent pathogen that could quickly spread across the world.^1-3^ The COVID-19 pandemic is that warning realized, and the full human and economic costs of the pandemic are still unknown. As of March 18, 2022, over 2 years after the World Health Organization (WHO) declared the COVID-19 outbreak a pandemic, there were over 460 million confirmed cases of COVID-19.^4^ It is estimated that the pandemic will result in as much as $12.5 trillion in losses to the global economy by 2024.^5^ In response, international financing institutions and other donors have allocated an unprecedented number of resources to mitigate the human and economic impacts of COVID-19.

All United Nations member countries are obligated to improve health security capacities under the conditions of the International Health Regulations (IHR) 2005 revision, a legally binding instrument developed by WHO that entered into force in 2007.^6^ The purpose of the IHR is “to prevent, protect against, control and provide a public health response to the international spread of disease.” While efforts have been made to formalize the specific activities that improve aspects of security within the IHR framework, interventions currently are not prioritized in order of relative importance for strengthening health security—especially in the context of responding to an ongoing public health emergency. Some evidence suggests that specific IHR capacities, such as legislation, coordination, and surveillance, could be associated with reduced COVID-19 incidence and mortality.^7^ There is still a lack of consensus, however, on the priority health system features that contribute to better pandemic preparedness and response that also contribute to larger progress toward universal health coverage.^7,8^ The large variance in the number of COVID-19 cases and deaths across countries with similar contexts suggests a dire need to identify priority investments, contextualize approaches to pandemic management, and encourage countries to strengthen capacity in areas that contribute to resilient health systems. Resilient health systems are those that are prepared for and can effectively respond to crises, maintain core functions when a crisis hits, and—informed by lessons learned during the crisis—can reorganize if necessary.^9,10^ Decisions on these investments can translate to better responses and improved performance during disease outbreaks, which could save thousands of lives.

International financing institutions are playing a vital role in filling financing gaps and providing needed support to governments. Commitments for COVID-19 response and recovery from multilateral development banks total $230 billion.^11^ This focus on health is unprecedented for many international financing institutions: for example, the Asian Development Bank's $20 billion package for COVID-19 response and recovery is nearly 4 times the cumulative amount that the bank has spent on health programs since 1990.^12^ While international financing institutions should be lauded for taking drastic measures to rapidly mobilize financing, there are gaps in the coordination of health security investments that potentially could result in fragmentation, duplication, and inequity in response. Interventions based on best practices need to be identified and prioritized to ensure that investments are effective in (1) reducing the immediate and protracted impact of COVID-19 and (2) strengthening health security. Additionally, these interventions should be documented and revisited to identify opportunities for strengthening capacities and systems to better prevent, detect, and respond to future health emergencies.

The evidence base for effective COVID-19 management is rapidly evolving. At the clinical level, the scientific community developed vaccines for prevention at record speed,^13,14^ evaluated and created new diagnostics for antigen and antibody detection,^15,16^ tested the impact of repurposed drugs for treatment,^17,18^ and created new, effective treatments.^19^ Modeling has demonstrated the benefits of nonpharmaceutical prevention measures such as physical distancing, use of face masks, case isolation, and lockdowns—when swiftly implemented—on COVID-19 mortality reduction.^20-23^ Despite emerging evidence for and against various interventions, little information exists on the enabling systemic conditions that maximize an intervention's impact, such as strengthened domestic laboratory networks or triage plans for treatment of patients during surge capacity. While guidance exists for national budgeting during the COVID-19 crisis, including essential functions such as budget prioritization, financial management, and strategic purchasing, there was little consensus on the priorities for international financing institutions to support.^24^ Development banks and other partners are well-positioned to finance these policy and systemwide interventions and should prioritize investments in COVID-19 response that will also contribute to strengthened health security, as defined and agreed upon by the global community.

Countries and financing partners continue to weigh difficult decisions and priorities in the COVID-19 crisis. Many high-income countries that were previously identified as among the most prepared for a pandemic faced significant challenges during the first months of COVID-19, which suggests there are gaps in the collective knowledge of effective pandemic mitigation and response.^25^ Until thorough analysis has identified the investments that contribute to resilient health systems, there is a need to rapidly but systematically prioritize investments to best use the resources made available for COVID-19 response.

In this article we provide guidance on systemwide interventions that effectively facilitate COVID-19 response, mitigation, and recovery while contributing to resilient health systems. To inform the allocation of resources during the COVID-19 pandemic response and circumstances that prevent in-person collaboration, we provide a “menu” of options and prioritized areas for investment, as agreed upon by health security experts from across the globe using a multistep interactive Delphi process.

## Methods

Given the need to provide rapid systematic guidance, under working constraints limited by lockdowns, we chose a modified Delphi technique to reach consensus on priority interventions for responding to COVID-19 and building resilient health systems. The Delphi process typically consists of a series of sequential questionnaires that seek to gain the most reliable consensus of opinion among a group of experts.^26,27^ Following best practices, a priori we described the objective of the study, the participant selection process, and the definitions of “consensus” (criterion to keep an intervention on the list) and “drop threshold (criterion for dropping an intervention from the list) and we limited the Delphi study to 2 rounds of questionnaires.^28^

We conducted the Delphi study to create a list of consensus interventions for responding to COVID-19 and building resilient health systems; the study comprised a series of steps between April 2 and May 15, 2020 (Figure). We first prepared an initial list of broader technical areas for investment and specific interventions based on a rapid literature review and consultations with key partners and stakeholders. The literature review consisted of assessments of IHR capacities and related documents,^6,29,30^ WHO COVID-19 action plans and regional disease control strategies,^31,32^ and academic publications; we chose to emphasize systematic reviews and supplements on lessons learned from IHR implementation and building resilient health systems.^33-36^ Following best practice methods for rapid literature reviews, we conducted a search of relevant published literature and limited the search by both date and language. One author selected studies and abstracted data into technical investment areas and interventions to strengthen these areas; the other author verified results and conducted the risk-of-bias assessment.^37^ We shared the findings from the literature review with an initial group of health security and financing experts from the Asian Development Bank, WHO, World Bank, and International Monetary Fund who further refined the list generated from the literature review.^38^ The initial list presented to Delphi study participants contained 11 technical areas and 37 specific interventions.

**Figure. Timeline for conducting the Delphi process in 2020:**
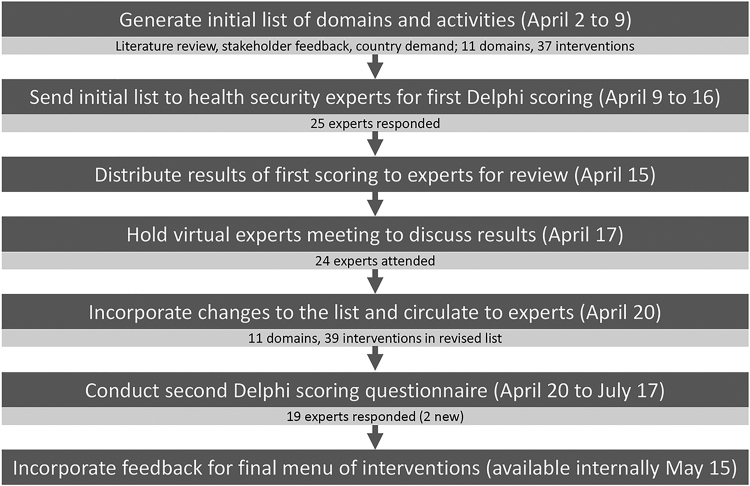
to determine priority areas for investment in COVID-19 response.

We identified 30 leaders in global health security based on authorship of influential publications and participation in professional working groups, with consideration for diversity of organizational representation. In all, 27 individuals from multilateral organizations, government, nongovernmental organizations, and academia participated in our study (Table 1). Along with an introduction to the broader exercise, the initial list of technical areas and interventions was distributed to the list of identified experts on April 9, 2020. We also requested that experts complete an online questionnaire, rating each of the broader technical areas for investment and specific interventions in terms of importance toward improving a country's ability to prevent, detect, and respond to COVID-19 and other disease outbreaks. Up to 3 personal correspondences via email and/or phone call were sent to experts before the teleconference to encourage a higher response rate. Experts scored each technical area and intervention on a 5-point Likert scale from 1 (“not at all important”) to 5 (“absolutely essential”). Additionally, respondents provided feedback on areas they felt were missing or required minor adjustment. Lastly, respondents were asked to participate in a virtual teleconference on April 17, 2020, to discuss the findings from this exercise and further refine the technical areas and interventions.

**Table 1.** Characteristics of Experts Participating in the Delphi Study

Category	*n* (%)
Sex	
Male	16 (59.3)
Female	11 (40.7)
Current organization	
Multilateral organization	9 (33.3)
Government	7 (29.2)
Academia	6 (22.2)
Nongovernmental organization	5 (18.5)
Geographic focus	
Asia-Pacific	15 (55.6)
Global	12 (44.4)

By April 14, 2020, 18 respondents had completed the online questionnaire. We circulated the preliminary results, specifically the percentage agreement on each technical area and intervention, to the entire expert group for their review in advance of the teleconference. The study team reviewed and synthesized unstructured feedback on reframing or adding new technical areas and interventions. On April 17, 2020, the virtual teleconference was convened and 24 experts attended from around the world. The entire group discussed the results and feedback from 25 total respondents (including the original 18 respondents). Within each technical area, specific interventions were presented in terms of whether they reached the consensus agreement threshold to stay on the list (at least 80% of respondents considering an intervention to be “very important” or “absolutely essential”) or to be dropped from the list (less than 50% of respondents considering an intervention to be “very important” or “absolutely essential”). In addition, broader topics within the technical area were discussed. Both the consensus agreement criterion and the drop threshold were defined a priori, based on best practice and on levels previously identified in the literature.^28,38^ Detailed minutes (ie, transcripts) from the meeting were reviewed and major themes were inductively coded and studied to determine emerging themes from the discussion.^39^

Following the teleconference, the study team incorporated feedback to revise the original list of technical areas and interventions for investment. We modified the wording of 3 technical areas and 8 interventions slightly for clarity and added 3 interventions to the list and dropped 1 intervention that did not meet the 50% threshold. On April 20, 2020, we circulated the resulting list of 11 technical areas and 39 interventions to experts and requested their participation in a second online questionnaire. As of May 13, 2020, 13 experts had responded to the survey, and the menu of consensus interventions based on expert responses was made available to Asian Development Bank staff. The second questionnaire remained open until July 17, 2020, and we sent up to 5 personal correspondences to experts to encourage responses. At the survey's closure, we received a total of 19 responses, 2 of which were from respondents who did not participate in the first round. In total, 27 experts participated in the Delphi questionnaires: 17 responded to both surveys, 8 responded to the first survey only, and 2 responded to the second survey only.

We analyzed the data using R version 3.6.3 (R Core Team, Vienna, Austria) and produced the figures using the package ggplot2.^40^ Percent agreement, mean, median, and standard deviation of scores were calculated and visualized for each technical area and intervention. Previous studies recommend ensuring stability between Delphi scoring rounds to determine that results are reliable.^38^ We used the Wilcoxon matched-pairs signed rank test to assess the stability of interventions among the 17 experts who responded to both rounds of Delphi scoring. We used the unpaired Wilcoxon signed rank test to assess the overall stability of interventions across all participants in the same round. Interventions were considered stable if there was no statistically significant difference in the average score of interventions between the first and second round at the alpha level of .05.

## Results

From the first round of Delphi scoring, all 11 technical areas achieved the consensus agreement criterion among participants (range from 80% to 100% agreement). Of the 37 specific interventions, experts reached consensus on 26 (70.3%) priority interventions for improving a country's ability to prevent, detect, and respond to COVID-19 and other disease outbreaks (Table 2). The percent agreement among interventions ranged from 40% to 100%. Building an animal health workforce was the only intervention that was dropped, as only 40% of respondents considered the intervention to be either very important or absolutely essential to COVID-19 response. The intervention that achieved the highest average score was “training health workers on infection prevention and control” (4.8 out of 5.0). Standard deviation of responses was generally low, ranging from 0.37 to 1.02.

**Table 2.** Results from Delphi Ranking Exercise

Domain	Activity	First Delphi Round (n = 25)				Second Delphi Round (n = 19)				Stability (*P *value)	
		Consensus (>3) %	Mean Rating	Median Rating	SD	Consensus (>3) %	Mean Rating	Median Rating	SD	Unpaired (*n*_1_ = 25, *n*_2_ = 19)	Paired (*n* = 17)
Surveillance and epidemiology^a^	Strengthening systems for contact tracing^b^	96%^c^	4.72	5	0.54	95%^c^	4.74	5	0.56	.86	.77
	Establishing enhanced surveillance systems (event, case-based, and/or environmental)^a,b^	84%^c^	4.28	5	0.94	95%^c^	4.68	5	0.58	.13	.01^e^
	Establishing national screening/referral guidelines and procedures^b^	88%^c^	4.48	5	0.71	84%^c^	4.21	4	0.71	.18	.04^e^
	Improving surveillance systems for zoonotic diseases	60%	3.60	4	1	84%^c^	4.26	4	0.73	.02^e^	.04^e^
	Supporting screening at ports of entry	72%	3.84	4	0.94	53%	3.53	4	0.96	.26	.67
Risk communication	Developing and testing messages and materials to be used for the COVID-19 outbreak on risk and potential impact of the pandemic	76%	4.12	4	0.88	95%^c^	4.21	4	0.54	.96	.79
	Developing and implementing information, guidelines, and training for healthcare professionals^b^	100%^c^	4.48	4	0.51	90%^c^	4.58	5	0.51	.53	.77
	Enhancing infrastructure to disseminate information from the national to subnational levels, and between the public and private sectors^b^	88%^c^	4.32	4	0.69	90%^c^	4.21	4	0.79	.72	.99
Policy and coordination mechanisms	Establishing/strengthening emergency operations centers or national incident management systems^b^	100%^c^	4.56	5	0.51	95%^c^	4.53	5	0.61	.99	.77
	Supporting countries to develop and implement national COVID-19 pandemic (and all hazards) prevention, preparedness, and response plans^a,b^	84%^c^	4.44	5	0.87	95%^c^	4.37	4	0.60	.37	.86
	Conducting epidemiologic and economic risk assessment and reduction/management plans^a,b^	84%^c^	4.32	4	0.75	95%^c^	4.47	5	0.61	.56	.42
	Establishing a vaccine preparedness plan^f^	–	–	–	–	95%^c^	4.47	5	0.61	n/a	n/a
	Establishing/strengthening national and/or regional One Health coordinating platforms^a^	60%	3.60	4	1	74%	4.11	4	0.81	.11	.04^e^
Social protection and social services^a^	Providing economic relief for those with reduced income	76%	4.24	4	0.83	100%^c^	4.74	5	0.45	.22	.23
	Ensuring a steady food supply for vulnerable populations^b^	92%^c^	4.52	5	0.65	100%^c^	4.68	5	0.48	.29	.42
	Providing schooling support for those with disrupted learning	64%	3.88	4	0.88	100%^c^	4.58	5	0.51	.17	.41
	Providing mental health and emotional support services^f^	–	–	–	–	95%^c^	4.21	4	0.71	n/a	n/a
Information systems	Sharing lessons learned^b^	92%^c^	4.44	5	0.77	100%^c^	4.42	4	0.51	.56	.99
	Improving health information systems^b^	80%^c^	4.16	4	0.75	84%^c^	4.16	4	0.69	.97	.99
	Integrating information systems across disciplines and nations	76%	4.04	4	0.84	74%	4.11	4	0.81	.86	.69
Hospital and primary healthcare capacity	Establishing guidelines for delivery of essential routine care^b^	84%^c^	4.32	4	0.75	95%^c^	4.53	5	0.61	.39	.48
	Upgrading existing facilities and expanding services^a^	72%	3.92	4	0.81	95%^c^	4.32	4	0.58	.10	.15
	Planning for triage facilities/nontraditional treatment sites during surges^b^	100%^c^	4.48	4	0.51	90%^c^	4.37	4	0.68	.71	.31
Health workforce	Strengthening community and clinical healthcare workforces^b^	96%^c^	4.48	5	0.59	90%^c^	4.53	5	0.70	.88	.20
	Enhancing response workforce capacities^f^	–	–	–	–	90%^c^	4.42	5	0.69	n/a	n/a
	Training epidemiologists^b^	84%^c^	4.12	4	0.67	84%^c^	4.16	4	0.83	.69	.99
	Building an animal health workforce^d,f^	40%	3.28	3	1.02	–	–	–	–	n/a	n/a
Building resilient health systems^a^	Supporting timely and flexible access to domestic emergency/crisis financing^b^	88%^c^	4.44	5	0.71	100%^c^	4.68	5	0.48	.29	.57
	Removing barriers to essential healthcare services^a,b^	92%^c^	4.56	5	0.65	95%^c^	4.68	5	0.58	.65	.77
	Removing financial barriers for COVID-19 patients^a^	72%	4.04	4	0.97	95%^c^	4.68	5	0.58	.02^e^	.04^e^
Diagnostic and lab capacity	Expanding domestic diagnostic capacity^b^	96%^c^	4.72	5	0.54	100%^c^	4.79	5	0.42	.78	.77
	Establishing/strengthening laboratory networks, surge plans, and information sharing at the subnational and regional levels^b^	100%^c^	4.64	5	0.49	100%^c^	4.68	5	0.48	.77	.48
	Procuring and/or producing essential diagnostic equipment and supplies^b^	96%^c^	4.72	5	0.68	95%^c^	4.74	5	0.56	.95	.99
	Strengthening laboratory staff capacity^b^	96%^c^	4.64	5	0.57	95%^c^	4.58	5	0.61	.74	.77
Community engagement	Enhancing local engagement^b^	92%^c^	4.48	5	0.65	100%^c^	4.63	5	0.50	.52	.78
	Supporting and incorporating feedback loops to decisionmaking^b^	92%^c^	4.28	4	0.74	84%^c^	4.37	5	0.76	.65	.76
	Mapping community networks and structures	72%	3.96	4	0.84	58%	3.89	4	0.88	.74	.78
Case management and infection prevention and control	Training health workers on infection prevention and control^b^	100%^c^	4.84	5	0.37	100%^c^	4.79	5	0.42	.68	.42
	Procuring PPE, oxygen delivery equipment, therapeutics, etc.^b^	96%^c^	4.8	5	0.50	100%^c^	4.79	5	0.42	.74	.73
	Adapting national treatment guidelines^a,b^	92%^c^	4.28	4	0.61	90%^c^	4.16	4	0.60	.51	.42

^a^ The wording of an intervention or technical area was adjusted between Delphi rounds (see Supplementary Table 1 for details).

^b^ Interventions that achieved consensus in both rounds.

^c^ Interventions that achieved consensus in a particular round.

^d^ An intervention was dropped.

^e^ An intervention was not stable across rounds.

^f^ An intervention was not included in a particular round. Abbreviations: PPE, personal protective equipment; SD, standard deviation.

Three key themes emerged from the participants during the teleconference: (1) risk communication and community engagement are complementary strategies and are pillars of any successful nonpharmaceutical intervention (eg, social distancing or contact tracing); (2) despite the COVID-19 crisis, ensuring that routine, essential services are maintained is a top priority to avoid additional outbreaks (eg, vaccine-preventable diseases) and ensuring that people have continued access to chronic care management; and (3) the COVID-19 pandemic has demonstrated the critical linkages between health, economics, and social inequality. Pandemic preparedness and response therefore requires all sectors to take responsibility and make contributions. Health interventions cannot be designed in isolation.

In addition to these themes, the discussion was useful in identifying current gaps in the proposed menu of interventions for COVID-19 response. From the initial feedback, 3 new interventions were proposed, namely (1) establishing a vaccine preparedness plan, (2) enhancing response workforce capacities, and (3) providing mental health and emotional support to those affected by COVID-19.

In the second round of Delphi scoring, all 11 technical areas reached consensus by the percent agreement criterion (range from 89% to 100% agreement). Further, participants reached consensus on 35 (89.7%) of the 39 interventions. No interventions were dropped in the second round. There were few additional comments in the second round online questionnaire and no interventions that required significant modification. The percent agreement on interventions in the second round ranged from 58% (1 intervention: mapping community networks and structures) to 100% (10 interventions). Expanding domestic diagnostic capacity, training health workers on infection prevention and control, and procuring medical supplies scored the highest on average across respondents (4.79 out of 5). Standard deviations ranged from 0.42 to 0.96.

Comparing the results from the 2 rounds of Delphi questionnaires gives insight on the reliability of changes to the menu of interventions and overall stability of findings. Across all interventions, the average percent agreement increased from 85% to 91% between rounds. Among interventions that were adjusted between the first and second round, an average increase of 11.6% in percent agreement was observed (ranging from a decrease of 3% to an increase of 23% in percent agreement). There were 9 more interventions that achieved the percent agreement consensus criterion in the second round. Stability was achieved for 34 interventions across all respondents using the unpaired Wilcoxon signed rank test (94.4% of the 36 interventions included in both rounds). Within individual respondents, 31 interventions achieved stability using the paired test (86.1% of interventions from both rounds). Among the 35 priority interventions that achieved consensus in the second round of scoring, 33 (94.2%) were considered to be stable by at least 1 measure.

## Discussion

For decades, public health experts and others have disputed the meaning of health security and resilient health systems, resulting in a broadly defined field.^41-43^ With COVID-19 causing a devastating loss of life and livelihood, governments and development partners have had to intervene to mitigate the effects of COVID-19 and strengthen health security without complete guidance on what actually contributes to a resilient health system. The menu of interventions presented in this article is a set of options to assist development partners and governments in prioritizing programming for COVID-19 resources. Structured discussions through the Delphi process enabled experts to make progress toward reaching consensus on key aspects of health security. While it is not a comprehensive strategy, the menu provides a starting point for countries and development partners to consider; as such, it can help set priorities for investing in capacities most relevant to the COVID-19 crisis and for preventing future disease outbreaks.

Across both rounds of the Delphi exercise, interventions to strengthen diagnostic and laboratory capacity, surveillance and epidemiology, and case management and infection prevention and control consistently reached consensus. This mirrors the “test, track, treat” strategy popularized by disease-specific control programs for malaria, HIV/AIDS, and tuberculosis.^44-46^ Early evidence from successful COVID-19 responses suggests that governments that are able to expand testing to understand community-level prevalence and incidence, conduct extensive contact tracing to cut off transmission chains, and successfully manage cases to prevent hotspots of infection were able to quickly “flatten the curve” of infection and secondary outbreaks in the early stages of the pandemic. South Korea and Vietnam are examples in Asia where these capacities have enabled effective responses. While technical areas related to test, track, and treat emerged as priorities, it is also vital that these interventions are not implemented in isolation.

A recurring theme from discussions with experts is that enabling conditions must be in place for traditional epidemiological interventions to be maximally effective. Strong political commitment from leadership to both control COVID-19 and build resilient health systems is a major facilitator of a successful response. Additionally, effective risk communication and community engagement initiatives must work in tandem to respond to community needs and ensure that public health measures are understood and followed. Strong buy-in from leadership is also essential: the open and consistent communication from Singapore's prime minister and government have been essential in maintaining public trust throughout the pandemic.^47^ Other enabling features, such as healthcare infrastructure, information systems, and other social services are also essential components of an effective response according to this exercise. The relative costs and benefits of individual interventions would be useful additions to this menu to further benefit decisionmakers. Future studies can conduct similar Delphi exercises with an emphasis on the cost-effectiveness, timeliness, and impact of these interventions and technical areas to further aid policymakers in the decisionmaking process.

Rigorous methods of monitoring and evaluation are needed to quantify the impacts of investments on preventing, detecting, and responding to public health emergencies. While this study has defined priority areas for investment, specific interventions within these topics must be further described and adapted to local contexts. Implementation research studies are necessary to understand how these interventions may contribute to improved health security.^48^

Validation studies should also be conducted by academic partners to further understand the relationships between capacities as defined in current measurement frameworks for health security (eg, the Joint External Evaluation^29^ or the State Party Self-Assessment Annual Reporting tool^49^) and their impact on COVID-19 mitigation. While these frameworks are comprehensive, they do not attempt to prioritize or suggest relative importance between health security capacities. Our study demonstrates that some interventions—such as mapping community networks, integrating information systems, and supporting screening at ports of entry—were not prioritized. Interventions related to One Health and animal workforces in particular were deemed to be of relatively low priority. This resulting prioritization could have been related to the composition of the experts involved in the Delphi study, but still may suggest that the One Health agenda is not sufficiently prioritized by health security experts. While there remains much work to be done, this study is an important first step in defining priority interventions for responding to COVID-19 and building resilient health systems to prevent future outbreaks.

## Conclusion

The findings from this study can apply to contexts globally, and interventions selected from the prioritized list should be tailored to meet the specific needs of countries and communities in which response is targeted. In addition to the findings, the study itself is a model for international collaboration during a crisis. Even under international lockdowns where interaction was limited to virtual meetings, the Delphi process provided a systematic and rapid methodology for generating evidence to guide substantial investments in health security. Feedback and participation from experts on the online Delphi process were overwhelmingly positive, and the experts were committed to the process as well as learning from each other. Results of this study have been used within the Asian Development Bank to inform project development for COVID-19 response and to continue building resilient health security systems in Asia and the Pacific.^50^ With new COVID-19 variants continuing to emerge and spread globally, it is important to improve detection and preparedness capacities while simultaneously maintaining response efforts. Through the application of these findings to future health investments, further research on the priority areas, and continued dedication from the public health community, Asia and the Pacific can be more resilient to future public health threats. More broadly, strengthening capacities and systems to better prevent, detect, and respond to future health emergencies benefits the entire global community.
